# Mesenchymal Stem Cells Exposed to Persistently High Glucocorticoid Levels Develop Insulin-Resistance and Altered Lipolysis: A Promising *In Vitro* Model to Study Cushing’s Syndrome

**DOI:** 10.3389/fendo.2022.816229

**Published:** 2022-02-24

**Authors:** Mariangela Di Vincenzo, Marianna Martino, Vincenzo Lariccia, Giulia Giancola, Caterina Licini, Giovanni Di Benedetto, Giorgio Arnaldi, Monia Orciani

**Affiliations:** ^1^Histology, Department of Clinical and Molecular Sciences, Università Politecnica delle Marche, Ancona, Italy; ^2^Division of Endocrinology and Metabolic Diseases, Department of Clinical and Molecular Sciences, Umberto I Hospital, Università Politecnica delle Marche, Ancona, Italy; ^3^Pharmacology, Department of Biomedical Sciences and Public Health, Università Politecnica delle Marche, Ancona, Italy; ^4^Clinic of Plastic and Reconstructive Surgery, Department of Experimental and Clinical Medicine, Università Politecnica delle Marche, Ancona, Italy

**Keywords:** glucocorticoids, MSCs, lipolysis, glucose uptake, insulin resistance

## Abstract

**Background:**

In Cushing’s syndrome (CS), chronic glucocorticoid excess (GC) and disrupted circadian rhythm lead to insulin resistance (IR), diabetes mellitus, dyslipidaemia and cardiovascular comorbidities. As undifferentiated, self-renewing progenitors of adipocytes, mesenchymal stem cells (MSCs) may display the detrimental effects of excess GC, thus revealing a promising model to study the molecular mechanisms underlying the metabolic complications of CS.

**Methods:**

MSCs isolated from the abdominal skin of healthy subjects were treated thrice daily with GCs according to two different regimens: lower, circadian-decreasing (Lower, Decreasing Exposure, LDE) *versus* persistently higher doses (Higher, Constant Exposure, HCE), aimed at mimicking either the physiological condition or CS, respectively. Subsequently, MSCs were stimulated with insulin and glucose thrice daily, resembling food uptake and both glucose uptake/GLUT-4 translocation and the expression of *LIPE*, *ATGL*, *IL-6* and *TNF-α* genes were analyzed at predefined timepoints over three days.

**Results:**

LDE to GCs did not impair glucose uptake by MSCs, whereas HCE significantly decreased glucose uptake by MSCs only when prolonged. Persistent signs of IR occurred after 30 hours of HCE to GCs. Compared to LDE, MSCs experiencing HCE to GCs showed a downregulation of lipolysis-related genes in the acute period, followed by overexpression once IR was established.

**Conclusions:**

Preserving circadian GC rhythmicity is crucial to prevent the occurrence of metabolic alterations. Similar to mature adipocytes, MSCs suffer from IR and impaired lipolysis due to chronic GC excess: MSCs could represent a reliable model to track the mechanisms involved in GC-induced IR throughout cellular differentiation.

## Introduction

Glucocorticoids (GCs) regulate a variety of physiological processes, such as metabolism, immune response, cardiovascular activity and brain function (1, 2). Chronic excess and dysregulation of GCs induces Cushing’s syndrome (CS), a complex clinical condition characterized by multisystem morbidities such as central obesity, hypertension, type 2 diabetes mellitus, insulin resistance (IR), dyslipidaemia, fatty liver, hypercoagulability, myopathy and osteoporosis (3–5). In patients with CS, GC secretion does not follow the circadian rhythm and consistently high serum GC levels are observed throughout the day (6, 7).

IR, defined as the reduced ability of insulin to control the breakdown of glucose in target organs, represents the common thread among obesity, metabolic syndrome and type 2 diabetes mellitus (8). GCs induce IR, but the mechanisms are complex and not completely understood. Under physiological conditions, the binding of insulin to its receptor on the cell surface induces the autophosphorylation of tyrosine in the insulin receptor substrate (IRS)-1 subunit with a consequent complex cascade of intracellular signals that leads to the inhibition of glycogen synthase kinase 3, the inhibition of apoptosis and the translocation of glucose transporter 4 (GLUT4) to the cell membrane with consequent glucose uptake (9, 10). Several studies have shown how chronic exposure to high levels of GCs reduces IRS-1 phosphorylation and protein expression, resulting in a lack of GLUT4 translocation and a reduction in glucose uptake in adipose tissue (11). In addition, the chronic excess of GCs increases lipoprotein activity and expression with subsequent release of circulating fatty acids, which, in turn, induce the phosphorylation of serine in IRS-1, thus compromising the mechanisms that lead to glucose transport into the cell (12).

In recent years, the involvement of mesenchymal stem cells (MSCs) in the onset of different pathologies has been addressed, and for some of them, MSCs have been identified as the real target for lasting therapeutic approaches (13, 14). MSCs are undifferentiated cells inside many tissues that are able to self-renew and differentiate into adipocytes, osteocytes and chondrocytes (15).

Adipose tissue, muscle tissue and bone are compromised in CS, so the involvement of MSCs in CS complications has been hypothesized; this was confirmed by our previous work reporting that MSCs isolated from the skin of patients affected by CS showed an altered wound healing process that is recognized as a clinical manifestation of CS (16).

In this scenario, it is tempting to speculate that the detrimental effects of excess GC could also affect MSCs, which may represent a promising cellular model to study the mechanisms leading to IR. The choice to use MSCs as a model is particularly interesting, since MSCs are the progenitors of mature adipocytes that may inherit and spread dysregulated mechanisms already present in MSCs.

Here, MSCs isolated from the abdominal skin of healthy subjects were treated *in vitro* with two different GC regimens, mimicking circadian cortisol rhythm and chronic hypercortisolism. Subsequently, cells were stimulated with insulin and glucose three times/day, resembling the normal uptake of food, and both glucose uptake and the expression of selected genes were analyzed to clarify the mechanisms underlying the development of IR and the occurrence of altered carbohydrate and lipid metabolism under chronic exposure to high levels of GCs.

## Materials and Methods

### Sample Collection

Seven abdominal skin samples were collected from healthy subjects (four males and three females age matched 42.3 ± 3.4) undergoing abdominoplasty at the Clinic of Plastic and Reconstructive Surgery, Università Politecnica delle Marche. Patients gave their informed consent; the study was approved by the Università Politecnica delle Marche Ethical Committee and conducted in accordance with the Declaration of Helsinki. The main demographical and clinical characteristics of enrolled patients are summarized in **Table 1**.

**Table 1:** Demographical and functional characteristics of enrolled patients.

Case	Age	Sex	Mean fasting glucose mmol/L	Mean glycosylated haemoglobin mmol/mol	Mean fasting insulin pmol/L	Mean total cholesterol mmol/L	Mean HDL cholesterol mmol/L	Mean triglycerides mmol/L
1	45	M	5.2	36	35.5	4.1	1.6	1.1
2	48	M	4.9	27	28.4	4.7	1.1	1.4
3	39	M	5.7	39	127.1	5	0.9	2
4	37	M	5.2	35	82.9	5.1	1.3	2
5	47	F	5.6	33	115.6	5.1	1.6	1.9
6	36	F	5.2	31	30.8	5	1.9	1.3
7	44	F	4.9	37	32.4	4.7	1.7	1.7
Mean	42		5.3	34	64.7	4.8	1.4	1.6
DS	±4		±0.2	±3	±43.2	±0.3	±0.3	±0.3

### Isolation and Characterization of MSCs

Cells were isolated from abdominal skin and then cultured with a Mesenchymal Stem Cell Growth Medium bullet kit (MSCGM, Lonza Group^®^ Ltd) as previously described (16) and characterized according to the criteria by Dominici (15). Plastic adherence, immunophenotype and multipotency were tested as already described (17–19). After the Oil Red staining, a semiquantitative analysis was carried out by dissolving the staining with 100% isopropanol and the absorbance was measured at 510nm in a microplate reader (Thermo Scientific Multiskan GO Microplate Spectrophotometer, Milano, Italy). In addition, the expression of *PPAR-γ* (peroxisome proliferator-activated receptor gamma) and *C/EBP-α* (CCAAT/enhancer-binding protein alpha) was tested by Real time PCR to confirm the adipocytes differentiation. Undifferentiated MSCs were used as control (C-MSCs). Briefly, after 21 days of culture in adipocytes differentiation medium, 2.5x10^5^ cells from the 7 patients were collected; cDNA synthesis and qRT–PCR were carried out as previously described (20). The primer sequences are summarized in **Table 2**. mRNA expression was calculated by the 2^−ΔΔCt^ method (21), where ΔCt=Ct (gene of interest)—Ct (control gene) and Δ (ΔCt)=ΔCt (differentiated MSCs)—ΔCt (undifferentiated MSCs). Genes were amplified in triplicate with the housekeeping genes *RPLP0* (Ribosomal Protein Lateral Stalk Subunit P0) and *GAPDH* (Glyceraldehyde-3-Phosphate Dehydrogenase) for data normalization. Of the two, *GAPDH* was the most stable and was used for subsequent normalization. The values of the relative expression of the genes are mean ± SD of three independent experiments.

**Table 2:** Primer sequences.

GENE	FORWARD 5’-3’	REVERSE 5’-3’
***GAPDH***	CCCTTCATTGACCTCAACTACATG	TGGGATTTCCATTGATGACAAGC
***RPLP0***	CCATTCTATCATCAACGGGTACAA	TCAGCAAGTGGGAAGGTGTAATC
***PPAR-γ***	AGCCTGCGAAAGCCTTTTGGTG	GGCTTCACATTCAGCAAACCTGG
***C/EBP-α***	AGGAGGATGAAGCCAAGCAGCT	AGTGCGCGATCTGGAACTGCAG
***LIPE***	AGCCTTCTGGAACATCACCGAG	TCGGCAGTCAGTGGCATCTCAA
***ATGL***	CCCACTTCAACTCCAAGGACGA	GCAGGTTGTCTGAAATGCCACC
***IL-6***	ATTCTGCGCAGCTTTAAGGA	AACAACAATCTGAGGTGCCC
***TNF-α***	CGAGTCTGGGCAGGTCTACTTT	AAGCTGTAGGCCCCAGTGAGTT

### Experimental Design: *In Vitro* Reproduction of Both Circadian Rhythm and Chronic Excess GCs and Food Uptake

**Figure 1:**
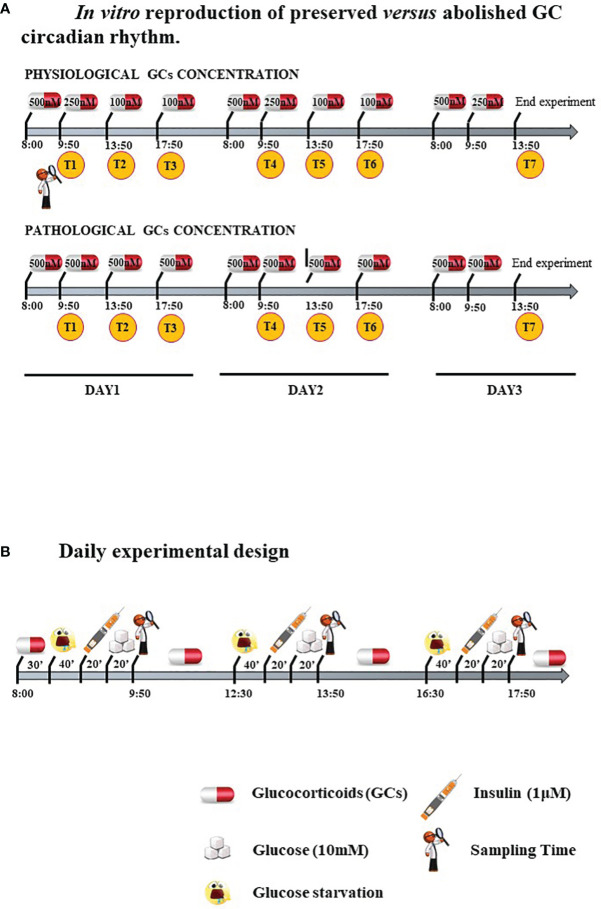
**(A)** *In vitro* reproduction of preserved *versus* abolished GC circadian rhythm. **(B)**. Daily experimental design.

Cells were treated with two different GC regimens: some were given lower, circadian-decreasing GC doses (Lower and Decreasing Exposure, LDE), some were exposed to persistently higher GC doses (Higher and Constant Exposure, HCE), to mimic *in vitro* either the preserved circadian rhythm or its pathologic abolishment in CS, as shown in **Figure 1A** and described in detail below. LDE cells were first exposed (8:00 a.m.-9:50 a.m.) to 500 nM hydrocortisone (MedChemExpress, MCE, Monmouth Junction, NJ, USA) and then to decreasing concentrations by replacing the medium with a fresh medium containing 250 nM hydrocortisone (9:50 a.m.-01:50 p.m.) and 100 nM (01:50 p.m.-05:50 p.m. and 05:50 p.m.-08:00 a.m.) of hydrocortisone (22). To mimic CS, HCE cells were exposed to 500 nM hydrocortisone for 24/24 hours. The 500 nM hydrocortisone medium was replaced with fresh medium at the same time as the physiological condition medium was changed.

Cells were starved and exposed three times/day to 10 mM glucose with or without prestimulation with 1 μM insulin (Sigma–Aldrich, Milano, Italy) to resemble daily food uptake.

Protocol is resumed in **Figure 1B**.

Cells derived from each single patient were divided into six experimental groups (Exp):

1) Exp 1, GLU: Cells exposed to glucose;

2) Exp 2, INS+GLU: Cells stimulated with insulin before glucose exposure;

3) Exp 3, LDE+GLU: LDE cells treated with glucose;

4) Exp 4, HCE+GLU: HCE cells treated with glucose;

5) Exp 5, LDE+INS+GLU: LDE cells stimulated with insulin before glucose exposure;

6) Exp 6, HCE+INS+GLU: HCE cells stimulated with insulin before glucose exposure.

In detail, cells were seeded in DMEM/F-12+10% FBS (Corning, NY, USA). After 24 hours, the medium was changed, and the cells were starved overnight with Advanced DMEM/F-12 w/o glucose (Lonza) with 0.5% FBS. At 8:00 a.m., starvation medium was replaced with a new medium containing hydrocortisone 500 nM for 30 minutes in groups exposed to GCs. After washing, the cells were glucose starved with KRPH buffer (20 mM HEPES, 5 mM KH2PO_4_, 1 mM MgSO_4_, 1 mM CaCl_2_, 136 mM NaCl and 4.7 mM KCl, pH 7.4) containing 2% BSA (Sigma–Aldrich) and hydrocortisone for 40 minutes. Cells from Exp 2, 5 and 6 were then stimulated with 1 μM insulin (Sigma–Aldrich) for 20 minutes. Finally, 10 mM glucose was added, and the time sampling was after 20 minutes.

The same protocol starting with starvation for 2 hours in DMEM/F-12 w/o glucose was repeated two times during the day, and the hydrocortisone concentration in the medium of LDE and HCE cells varied accordingly.

To evaluate the long-term impact on metabolism and IR, the experiment was performed for three days with repeated sampling times after glucose administration: T1, T2 and T3 at 9:50 a.m., 1:50 p.m., 5:50 p.m. of the first day; T4, T5 and T6 at 9:50 a.m., 1:50 p.m., 5:50 p.m. of the second day; T7 at 1:50 p.m. of the third day (**Figure 1A**).

The entire experiment (Exp 1-6, from T1 to T7) was repeated thrice, and data are reported as mean± standard deviation (SD) over the three independent experiments.

### XTT Assay

To evaluate whether repeated starvation steps and treatments would affect cell viability and consequently influence the measurement of glucose uptake, an XTT assay (Sigma–Aldrich) was initially performed. A total of 3x10^3^ cells/well belonging to Exp 1, 2, 4 and 6 derived from the 7 patients were plated in a 96-well plate and treated as previously described. Another experimental group was included as a control, consisting of cells continuously cultured in starvation medium (STARVED CTRL). The XTT assay was performed at the end of each day (T3, T6 and T7 sampling times) following the manufacturer’s instructions. The experiment was repeated thrice, and data are reported as mean ± SD over the three independent experiments.

### MSCs Responsiveness to Insulin

To evaluate whether MSCs were responsive to insulin, glucose uptake and the cellular localization of GLUT4 were first evaluated in MSCs not treated with GCs (Exp 1 and 2) from T1 to T6.

For the glucose uptake assay, 3x10^3^ cells/well were plated in a 96-well plate and treated according to the above protocol; after insulin stimulation, 10 mM of 2-deoxyglucose (2-DG) was added for 20 minutes, and a colorimetric assay was performed following the manufacturer’s instructions. The readings were at 420 nm in a microplate reader (Thermo Scientific Multiskan GO Microplate Spectrophotometer, Milano, Italy).

For the cellular distribution of GLUT4, 1.5x10^4^ cells (Exp 1 and 2 derived from the 7 patients) were seeded in triplicate on coverslips and treated as indicated before until T5 sampling time. Cells were then washed, fixed with 4% PFA and permeabilized for 30 min. Subsequently, cells were incubated with anti-GLUT4 antibody (Santa Cruz Biotechnology, USA) followed by treatment for 30 min with a goat anti-mouse FITC-conjugated antibody (23). Finally, coverslips were mounted on glass slides in Vectashield (Vectorlabs, CA, USA), and confocal imaging was performed using a Zeiss LSM510/Axiovert 200 M microscope with an objective lens at 20× magnification (24). Line scans were acquired excluding nuclear regions, and GLUT4 immunofluorescence was analyzed as described elsewhere.

### Effects of Different GC Regimens on Glucose Uptake and GLUT4 Translocation

After having proven that MSCs could function as a cellular model, since they were responsive to insulin, the potential effects of both GC regimens on glucose uptake were evaluated.

Glucose uptake was measured in the experimental groups treated with GCs (Exp 3, 4, 5 and 6 derived from the 7 patients), and GLUT4 translocation was evaluated in cells from Exp 4 and 6 as described above.

### Expression of Genes Involved in the Development of IR

The expression of selected genes, such as *LIPE*, *ATGL*, *IL-6* and *TNF-α* (coding for hormone-sensitive Lipase E, Adipose TriGlyceride Lipase, InterLeukin-6 and Tumour Necrosis Factor-α, respectively), was evaluated to clarify the mechanisms involved in the development of IR in MSCs (25–28). A total of 2.5x10^5^ cells/well belonging to Exp 5 and 6 from the 7 patients were seeded in triplicates in a 6-well plate and treated following the experimental design. Pellets were collected at T2 and T7, which were chosen as sampling times representing acute and chronic exposure to GCs. RNA extraction, cDNA synthesis and qRT–PCR were carried out as previously described (20). The primer sequences are summarized in **Table 2**. mRNA expression was calculated by the 2^−ΔΔCt^ method (21), where ΔCt=Ct (gene of interest)—Ct (control gene) and Δ (ΔCt)=ΔCt (HCE+INS+GLU)—ΔCt (LDE+INS+GLU). All selected genes were amplified in triplicate with the housekeeping genes *RPLP0* and *GAPDH* for data normalization. Of the two, *GAPDH* was the most stable and was used for subsequent normalization. The values of the relative expression of the genes are mean ± SD of three independent experiments.

### Statistical Analysis

For statistical analysis, GraphPad Prism 6 Software was used. All data are expressed as the mean ± standard deviation (SD). For parametric analysis all groups were first tested for normal distribution by the Shapiro–Wilk test (29) and comparison between 2 groups were performed by unpaired Student’s t test. For two-sample comparisons, significance was calculated by unpaired t-Student’s test while the ordinary one-way ANOVA test was used for multiple comparison (Tukey’s multiple comparisons test). Significance was set at p value < 0.05.

## Results

### MSCs Isolation and Characterization From Abdominal Skin

MSCs isolated from abdominal skin appeared homogeneous with a fibroblastoid morphology and showed adherence to plastic. According to Dominici’s criteria (17), cells were positive for CD73, CD90 and CD105, and negative for HLA-DR, CD14, CD19, CD34 and CD45.

**Figure 2:**
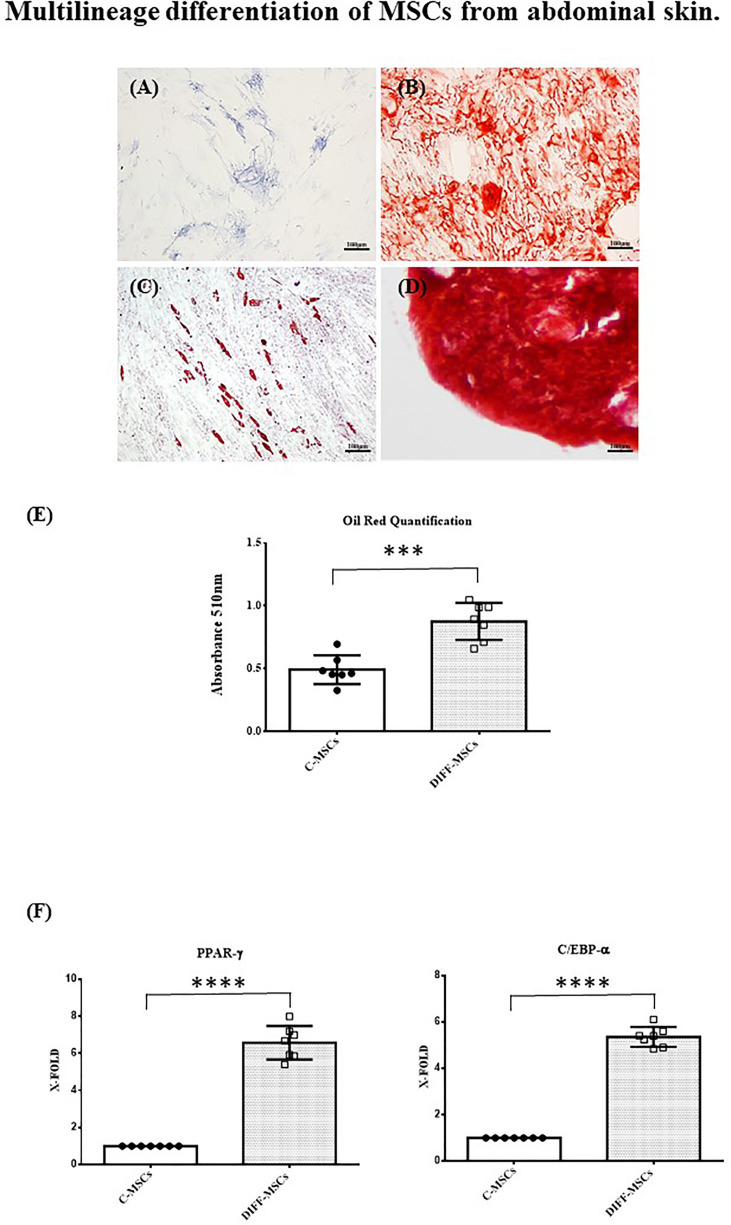
Multilineage differentiation of MSCs from abdominal skin. Representative images of MSCs derived from the seven patients and differentiated towards osteogenic lineage as assessed by ALP reaction (**A**, Scale bar 100μm) and Alizarin red staining (**B**, Scale bar 100μm); chondrogenic lineage as indicated by Safranin-O staining (**C**, Scale bar 100 μm); adipocyte lineage as confirmed by Oil red staining (**D**, Scale bar 100μm); **(E)** Oil Red staining quantification. Data are expressed as mean ± SD of the absorbance read for undifferentiated and differentiated cells (C-MSCs and DIFF-MSCs respectively). **(F, G)** Expression of *PPAR-γ* and *C/EBP-α* by RT-PCR in differentiated vs undifferentiated MSCs towards adipogenic lineage. Data are expressed as mean ± SD (over three independent experiments) of the X-fold (2^−ΔΔCt^ method) of differentiated MSCs compared to undifferentiated MSCs, arbitrarily expressed as 1, where ΔCt=Ct (gene of interest)—Ct (control gene) and Δ (ΔCt)=ΔCt (DIFF-MSCs)—ΔCt (C-MSCs). Unpaired t-Student’s test; ***p<0.001, ****p<0.0001.

Cells were also able to differentiate towards osteogenic, chondrogenic and adipogenic lineages. After 7 days of osteogenic differentiation, cells showed alkaline phosphatase activity (**Figure 2A**), and after 14 days, cells were strongly positive for alizarin red staining (**Figure 2B**). Chondrogenic differentiation was achieved after 30 days, as shown by safranin-O staining (**Figure 2C**). MSCs differentiation into adipocytes occurred after 21 days, as evidenced by the presence of lipid vacuoles after oil red staining (**Figure 2D**). Its quantification confirmed as the amount of lipid vacuoles was higher in differentiated cells than in control cells (C-MSCs; **Figure 2E**). The expression of *PPAR-γ* and *C/EBP-α* was tested after 21 days of culture in differentiating medium and it was higher in differentiated than in undifferentiated MSCs (**Figures 2F, G**).

### Cell Viability by XTT Assay

**Figure 3:**
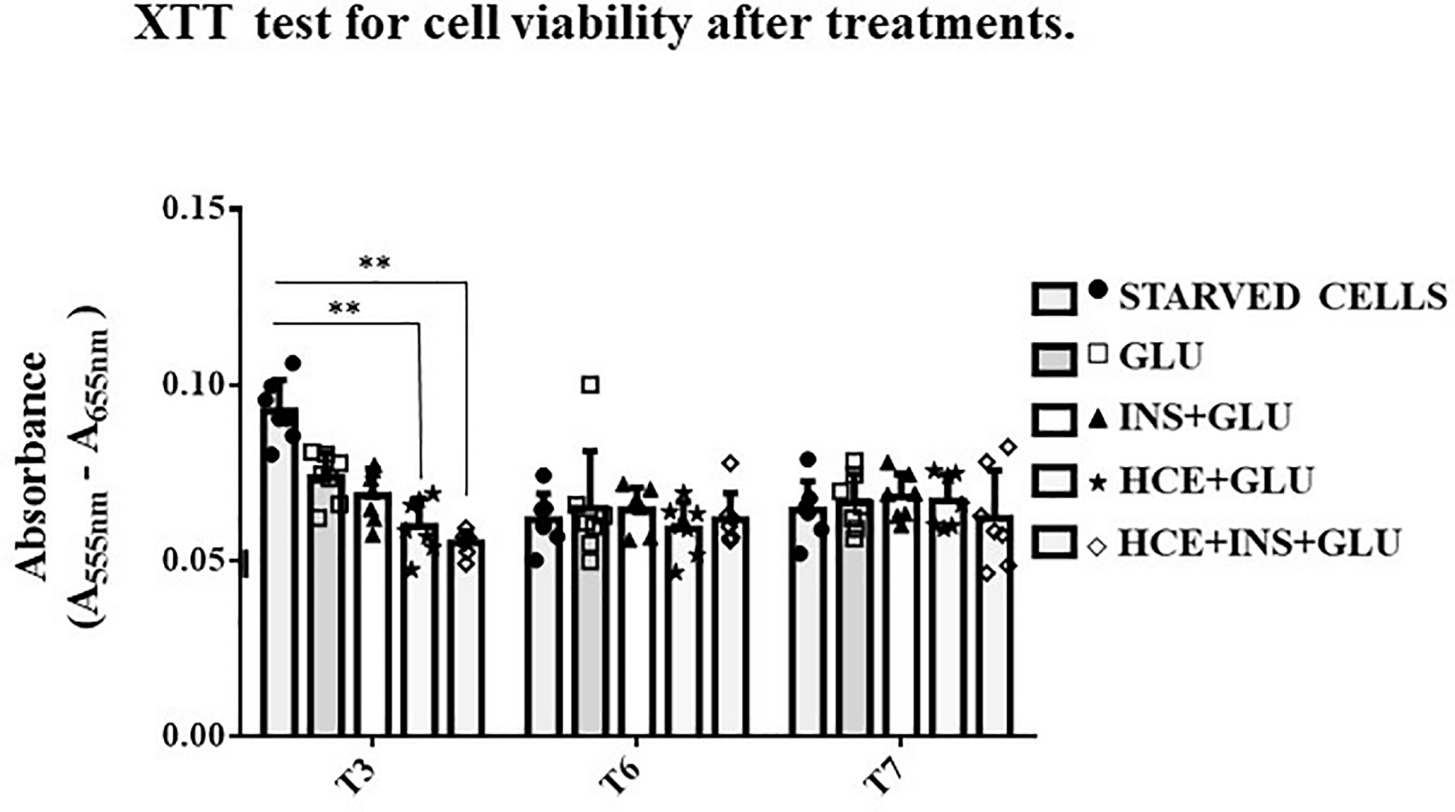
XTT test. The bars indicate cells’ viability at T3, T6 and T7 sampling times. One-way ANOVA; **p < 0.01 vs STARVED CTRL inside each time sampling. STARVED CTRL: cells continuously cultured in starvation medium; GLU: Cells exposed to glucose; INS+GLU: Cells stimulated with insulin before glucose exposure; HCE+GLU: HCE (Higher and Constant Exposure) cells treated with glucose; HCE+INS+GLU: HCE cells stimulated with insulin before glucose exposure. Data are expressed as mean ± SD of the absorbance read for MSCs derived from each single patient over three independent experiments.

**Figure 3** shows that the viability of the STARVED CTRL (cells continuously cultured in starvation medium) was significantly increased compared to that of the HCE cells at T3 but not thereafter. Although repeated interventions caused a proliferation block earlier than starvation alone, the different treatments did not interfere with vitality, and further analyses on glucose uptake were unaffected by different cell mortality during the experiment.

### MSCs Responsiveness to Insulin

**Figure 4:**
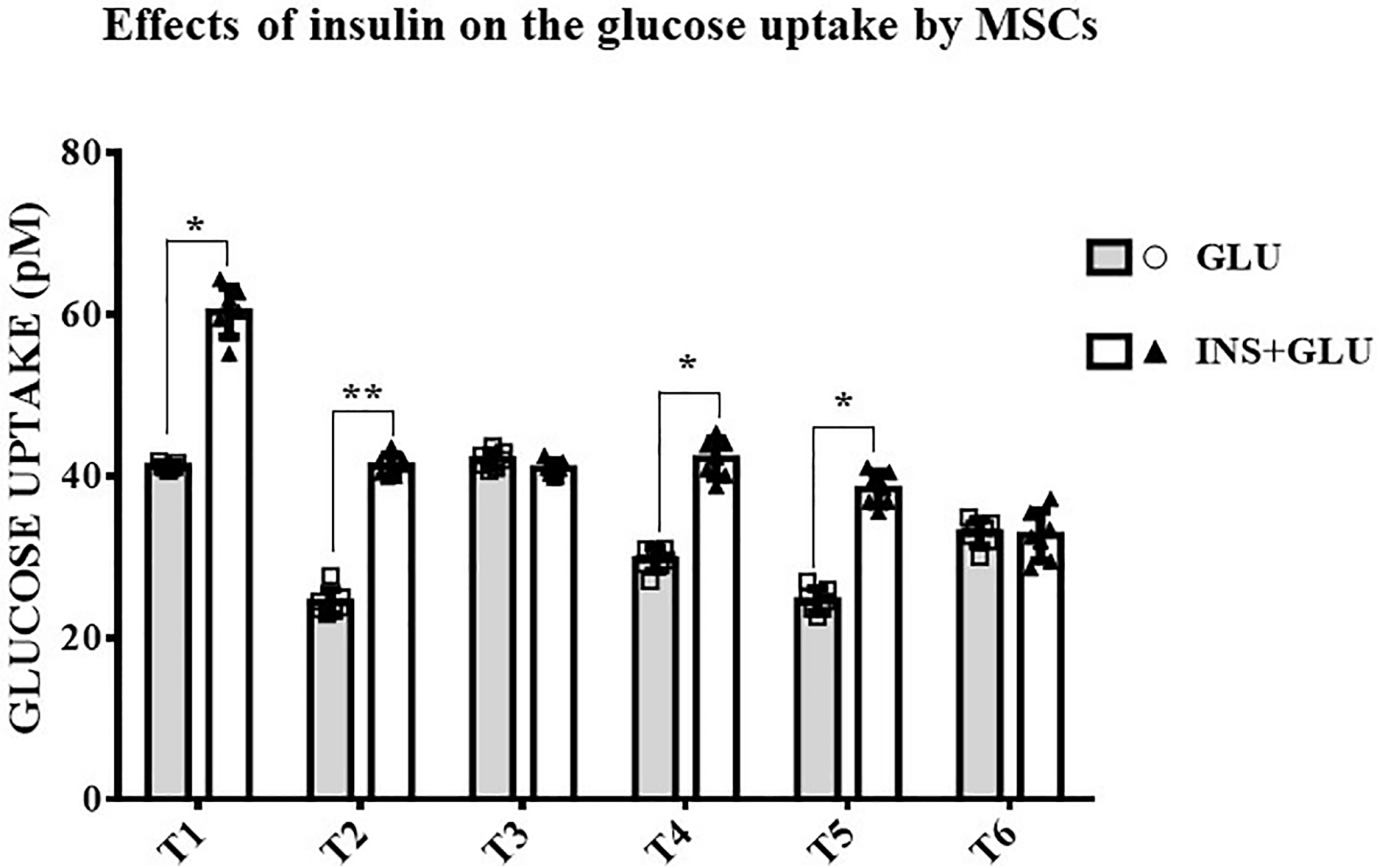
Responsiveness of MSCs to insulin. The bars show the glucose uptake expressed in pM at T1, T2, T3, T4, T5 and T6 in insulin-stimulated or non-stimulated MSCs. Unpaired t-Student’s test; *p < 0.05, **p < 0.01. GLU: Cells exposed to glucose; INS+GLU: Cells stimulated with insulin before glucose exposure. Data are expressed as mean ± SD of the readings for MSCs derived from each single patient over three independent experiments.

As shown in **Figure 4**, stimulation with insulin significantly increased glucose uptake at T1, T2, T4 and T5, whereas at T3 and T6, the level of glucose uptake did not differ significantly between insulin-treated (Exp2, INS+GLU) and nontreated (Exp1, GLU) cells.

**Figure 5:**
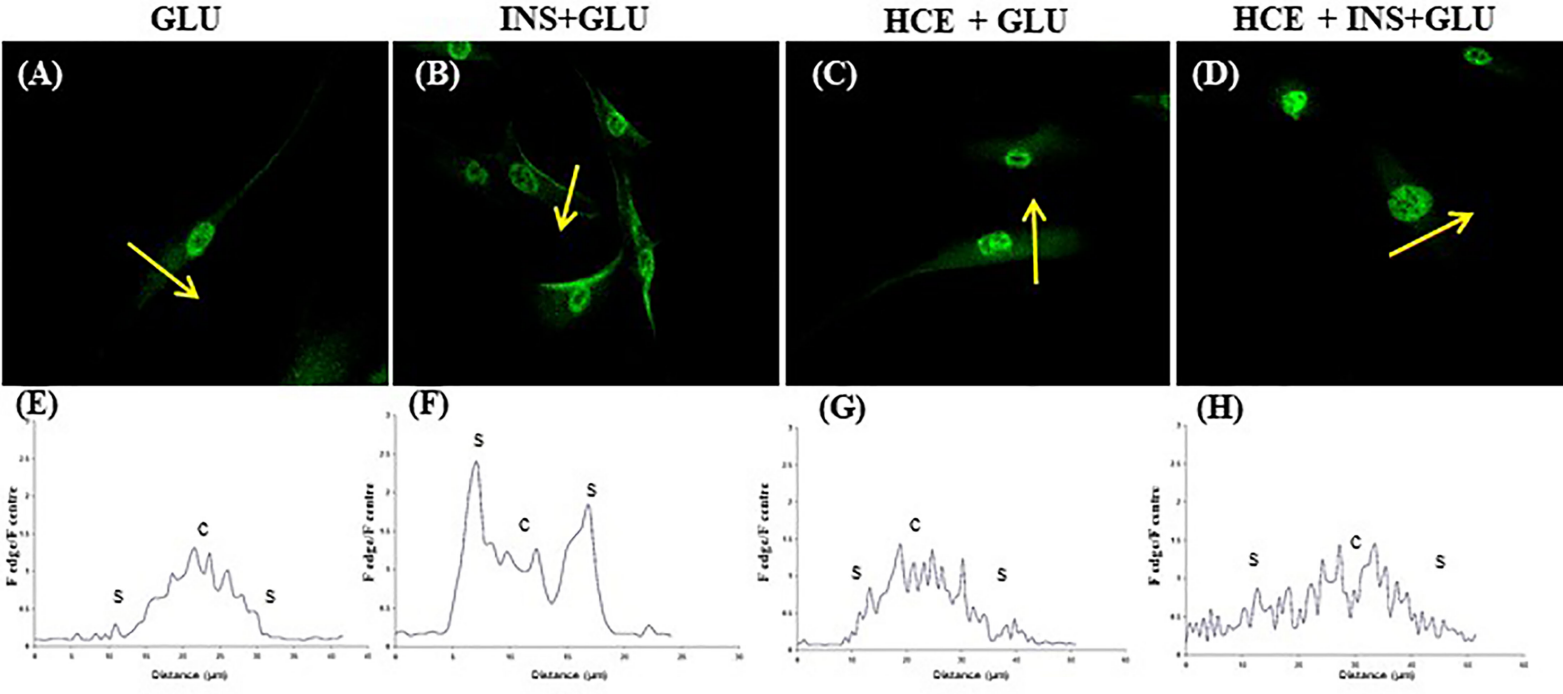
GLUT4 translocation. Representative confocal images of GLUT4 in MSCs derived from the seven patients and stimulated **(B, D)** or not **(A, C)** with insulin and exposed to 500nM of GCs **(C, D)**. The graphs **(E–H)** show the fluorescence ratio between the edge and the centre of the cell; yellow arrows indicate the portion of cell subjected to analysis. GLU: Cells exposed to glucose; INS+GLU: Cells stimulated with insulin before glucose exposure; HCE+GLU: HCE (Higher and Constant Exposure) cells treated with glucose; HCE+INS+GLU: HCE cells stimulated with insulin before glucose exposure.

Notably, in the absence of insulin, GLUT4 was more localized in the perinuclear area of the cells (**Figures 5A, E**). Insulin stimulation enhanced GLUT4 translocation towards the plasma membrane (**Figures 5B, F**).

### Effects of LDE and HCE on GCs on Glucose Uptake and GLUT4 Translocation

**Figure 6:**
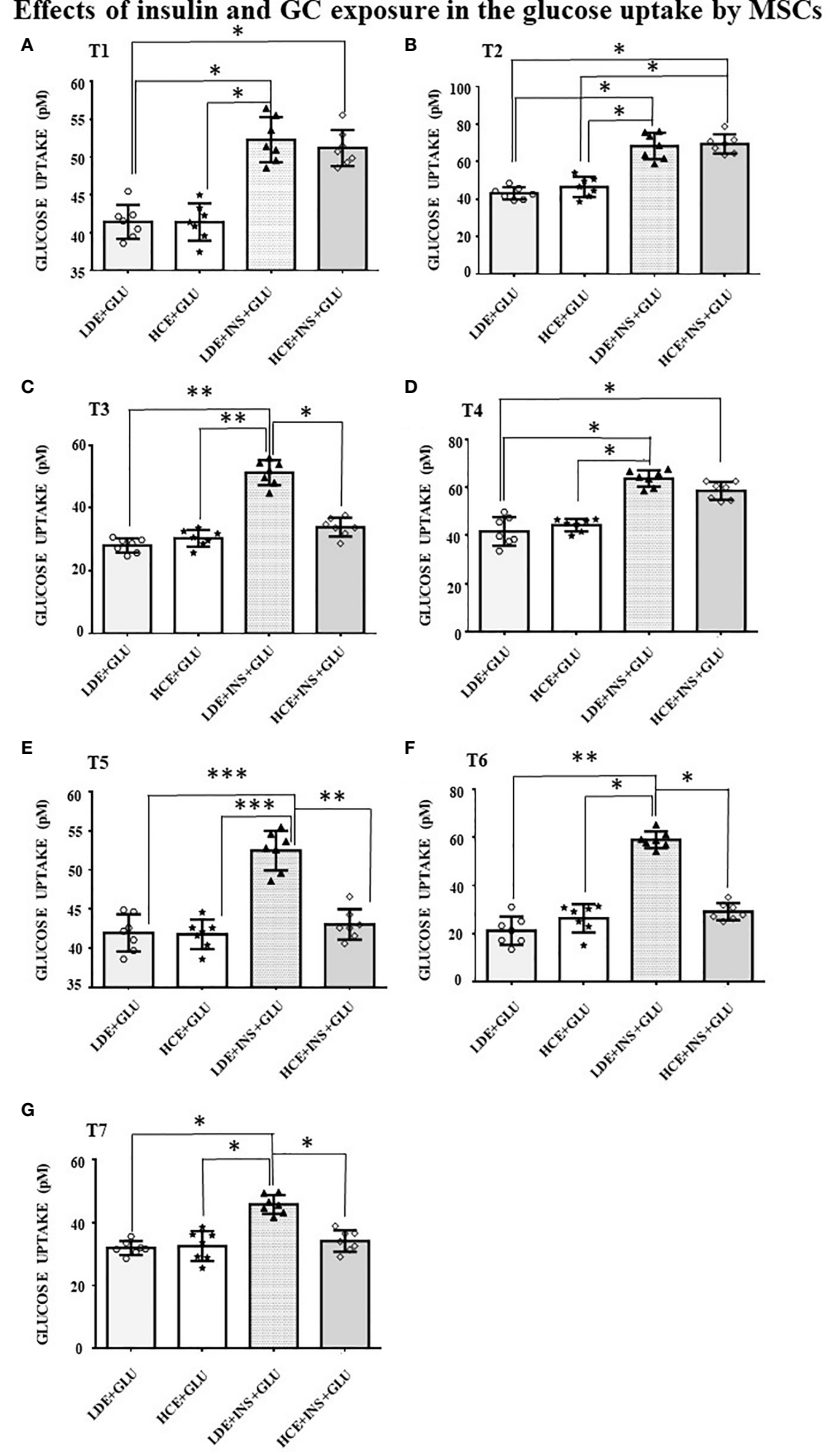
Glucose uptake in MSCs undergoing a LDE or a HCE to GCs. The bars represent the glucose uptake expressed in pM at T1 (9:50 a.m. first day, **A**), T2 (1:50 p.m. first day, **B**), T3 (5:50 p.m. first day, **C**), T4 (9:50 a.m. second day, **D**), T5 (1:50 p.m. second day, **E**), T6 (5:50 p.m. second day, **F**) and T7(1:50 p.m. third day, **G**) in MSCs undergoing a LDE or a HCE to GCs and stimulated or not with insulin. One-way ANOVA; *p < 0.05,**p < 0,01,***p < 0,001. LDE+GLU: LDE (Lower and Decreasing Exposure) cells treated with glucose; HCE+GLU: HCE (higher and Constant Exposure) cells treated with glucose; LDE+INS+GLU: LDE cells stimulated with insulin before glucose exposure; HCE+INS+GLU: HCE cells stimulated with insulin before glucose exposure. Data are expressed as mean ± SD of the readings for MSCs derived from each single patient over three independent experiments.

In LDE cells, insulin induced a significant increase in glucose uptake at all sampling times (**Figure 6**). Conversely, GC administration did not interfere with glucose uptake by HCE cells in the acute period (T1, T2) but led to a significant decrease in glucose uptake when prolonged (T3, T5, T6, T7). Accordingly, GLUT4 translocation was inhibited irrespective of insulin stimulation (**Figures 5C, G** and **D, H**) in HCE cells.

### Effect on Lipolysis and Development of IR: Gene Expression

**Figure 7:**
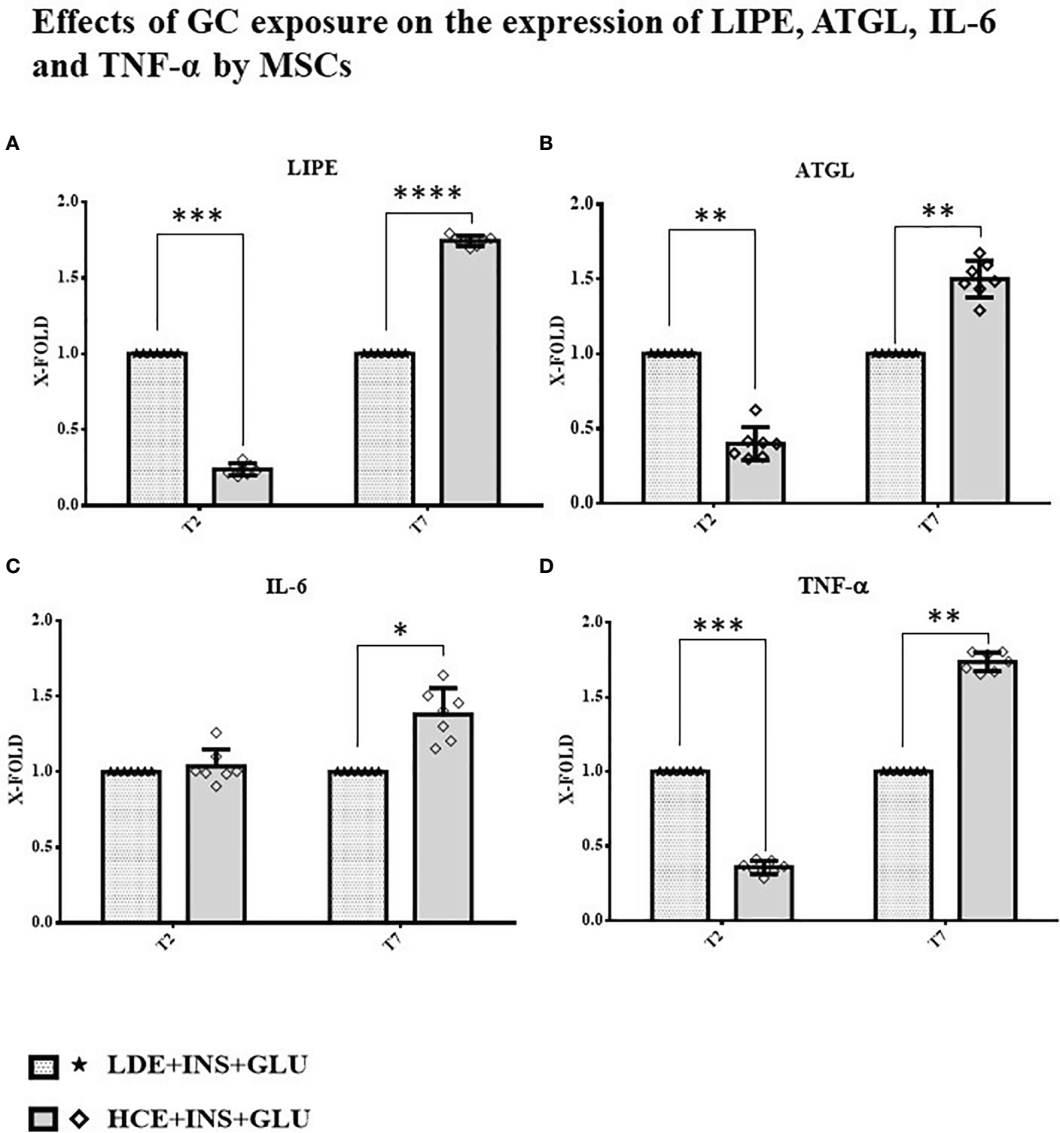
Gene expression in MSCs undergoing a LDE or a HCE to GCs. The bars display the expression of genes referred specifically to the development of IR: **(A)**: *LIPE*, **(B)**: *ATGL*, **(C):** *IL-6* and **(D):** *TNF-α* at T2 and T7 sampling times. LDE+GLU+INS: LDE (Lower and Decreasing Exposure) cells stimulated with insulin before glucose exposure; HCE+GLU +INS: HCE (higher and Constant Exposure) cells stimulated with insulin before glucose exposure. Data are expressed as mean ± SD (over three independent experiments) of the X-fold (2^−ΔΔCt^ method) of HCE+INS+GLU compared to LDE+INS+GLU arbitrarily expressed as 1, where ΔCt=Ct (gene of interest)—Ct (control gene) and Δ (ΔCt)=ΔCt (HCE+INS+GLU)—ΔCt (LDE+INS+GLU). Unpaired t-Student’s test; *p < 0.05,**p < 0.01,***p < 0.001;****p < 0.0001.

A downregulation of both genes involved in the breakdown of triglycerides to fatty acids (*LIPE* and *ATGL*) was found at T2, whereas at T7, their expression was significantly increased in HCE cells compared to LDE cells. At T7, HCE cells showed a significant increase in the expression of both IL-6 and TNF-α genes, whereas at T2, only the expression of TNF-α was lower than that of LDE cells (**Figure 7**).

## Discussion

The clinical presentation of CS is well established, but the mechanisms underlying the onset of some of its complications, IR above all, have not yet been fully understood and may involve tissue-specific players. As progenitors of specialized cellular lines that are directly implicated in the progression of chronic GC excess-induced damage (such as adipocytes, skeletal muscle cells and osteocytes), MSCs are of particular interest: in a previous study, we showed that MSCs derived from the skin of patients with CS displayed dysregulated inflammatory markers and altered expression of genes related to wound healing, demonstrating not only how they could be a useful cellular model to study this event but also their potential contribution to the development of CS manifestations (16).

With this premise, we hypothesized that MSCs exposed to excess GC encounter altered glucose uptake mechanisms, which are then inherited and consolidated by their derived, specialized cells.

Our work aimed to explore and compare the effects of two different GC regimens (LDE- Lower and Decreasing Exposure- and HCE- Higher and Constant Exposure) on glucose and lipid metabolism in MSCs.

First, MSCs were isolated from abdominal skin and characterized by confirming their undifferentiated state (15). To faithfully reproduce the circadian variations in GC concentrations and food intake, cells were treated by following an articulated protocol (**Figure 1**).

It is well established that insulin stimulation promotes glucose uptake *via* GLUT4 translocation (30–32) in adipocytes and skeletal muscle cells, but the same mechanism has not yet been demonstrated for MSCs. Therefore, the responsiveness of MSCs to insulin, as well as the involvement of GLUT4 in glucose uptake, were addressed before evaluating the effects of GCs. We demonstrated that the exposure of MCSs to insulin increased their glucose uptake and insulin-induced GLUT4 translocation with mechanisms that are similar to those described for adipocytes and muscle cells by confocal imaging. In contrast to what was previously reported for adipocytes (33, 34), GLUT4 expression before insulin stimulation occurred in the cytoplasmic, perinuclear and nuclear compartments in a nonvacuolized pattern. The same localization was observed by Tonack et al. in mouse embryonic stem cells (35). As in adipocytes, the protein translocated on the cell surface, favoring glucose uptake after insulin stimulation.

These results opened the second part of the research aimed at evaluating the IR-inducing effects of GCs on MSCs.

MSCs were exposed to two different GC regimens: in LDE cells, insulin stimulation always caused an increase in glucose uptake, confirming that insulin sensitivity of MSCs is not altered when cortisol circadian rhythm is preserved; conversely, in HCE cells, an impaired response to insulin was observed, as demonstrated by their decreased glucose uptake. These observations were also confirmed by confocal data, showing how excess GC blocked the insulin-induced translocation of GLUT4 from the intracellular compartment to the cell surface. Of note, a reduction in glucose uptake was not detected in earlier sampling times (T1, T2) but later (T3, T5, T6, T7). These results, taken together with the lack of GLUT4 translocation, suggest that IR develops over time. The development of IR following chronic exposure to GCs has been widely demonstrated in differentiated cells such as adipocytes, hepatocytes, muscle and endothelial cells (36–38), but to our knowledge, this has never been observed in human stem cells before.

Our results are in line with those by Gathercole et al. (12), who reported increased insulin-stimulated glucose uptake in a human immortalized subcutaneous adipocyte line (Chub-S7) after acute exposure to dexamethasone, as well as to hydrocortisone (up to 48 hours, in a dose- and time-dependent manner for the latter), thus proposing that the development of GC-induced obesity was promoted by enhanced adipocyte differentiation. However, it must be noted that although Chub-S7 are not fully differentiated adipocytes, they cannot be considered MSCs.

In our study, MSCs showed transient signs of IR at T3. In our opinion, this finding represents a physiologic phenomenon and is in line with previous findings in healthy volunteers who were administered hydrocortisone at two different time points and whose endogenous cortisol production was suppressed by metyrapone and nutrient intake was controlled by means of a continuous glucose infusion (39): subjects receiving hydrocortisone in the evening showed a more pronounced delayed hyperglycaemic effect than those taking hydrocortisone in the morning (39). Persistent signs of IR in our MSCs appeared even earlier (from T5, after 30 hours of HCE to GCs) than Gathercole’s Chub-S7 (12): the ability of MSCs to develop early documentable and conceptually plausible alterations, which can be tracked even once differentiated, further confirms that they are a reliable model for physiopathology studies.

The relationship between insulin and lipolysis is bidirectional: inhibition of lipolysis is mainly due to insulin (24), but different mechanisms have been identified where increased lipolysis is involved in the impairment of insulin sensitivity (25, 40). Boden et al. (41) reported that increasing circulating nonesterified fatty acid (NEFA) levels by lipid infusion induced transient IR. To obtain a clearer picture of the possible mechanisms involved in the development of IR in MSCs, we analyzed the expression of *LIPE* and *ATGL* genes at different timepoints. We found that HCE cells showed an initial reduction (T2), followed by a significant increase (T7), in the expression of *LIPE* and *ATGL* genes compared to LDE cells. The results from previous works on this topic are partially conflicting: Slavin (42) and Villena (43) found upregulated expression of the *LIPE* and *ATGL* genes, respectively, after a short treatment with GCs, but studies examining the effects of prolonged GC administration suggested that the acute induction of systemic lipolysis by GCs was not sustained over time (44). However, in these *in vitro* studies, cells were never treated with insulin, whose counterregulatory effect on lipolysis could not be highlighted. Notably, diabetic patients with CS show an increased activation of lipolysis due to IR (44). Our results fully reflect this scenario, showing that the lipolytic effects are even more marked once insulin levels fail to compensate for associated IR. *LIPE* and *ATGL* gene expression was downregulated at T2, when IR had not yet been reached; at T7, when chronic exposure to high GC levels compromised insulin sensitivity, both lipolysis-related enzymes were overexpressed. Of note, increased expression of *LIPE* and *ATGL* genes in the presence of IR was also reported by Sumuano et al. in mature adipocytes (37). Given its ability to decrease the tyrosine kinase activity of the insulin receptor, TNF-α is an important mediator of IR in obesity and type 2 diabetes mellitus (26). IL-6 is notably associated with IR by both sustaining low-grade chronic inflammation (45) and impairing the phosphorylation of insulin receptor and IRS-1 (27). In agreement with these statements, *TNF-α* and *IL-6* expression was lower before IR induction (T2) and higher after prolonged exposure (T7) in HCE cells than in LDE cells, further confirming the importance of preserved circadian GC rhythmicity to prevent the occurrence of metabolic alterations.

## Conclusions

MSCs derived from skin could be a good human model for studying the toxic effects of GCs. Like mature adipocytes, they are responsive to insulin stimulation that promotes glucose uptake *via* GLUT4 translocation, and their chronic exposure to excessive levels of GCs induces the development of IR. For differentiated cells, impaired lipolysis is observed in MSCs once IR has arisen. Furthermore, MSCs could be a promising model to track the mechanisms involved in GC-induced IR throughout cellular differentiation. Functional analyses will be necessary to elucidate the mechanisms behind these first descriptive results and overcame the actual weakness of this research. In addition, co-cultures with MSCs and mature adipocytes will be performed to investigate the crosstalk between these two cell types.

## Data Availability Statement

The original contributions presented in the study are included in the article/supplementary material, further inquiries can be directed to the corresponding author.

## Ethics Statement

The studies involving human participants were reviewed and approved by Università Politecnica delle Marche Ethical Committee. The patients/participants provided their written informed consent to participate in this study.

## Author Contributions

Conceptualization, MO and GA. Methodology, MDV and MM. Formal analysis, MDV, VL, and CL. Data curation, GDB and GG. Writing—original draft preparation, MO and MDV. Writing—review and editing, MO, GA, and MM. Supervision, MO and GA. All authors have read and agreed to the published version of the manuscript.

## Funding

This work was supported by 2017HRTZYA_005 project grant.

## Conflict of Interest

The authors declare that the research was conducted in the absence of any commercial or financial relationships that could be construed as a potential conflict of interest.

## Publisher’s Note

All claims expressed in this article are solely those of the authors and do not necessarily represent those of their affiliated organizations, or those of the publisher, the editors and the reviewers. Any product that may be evaluated in this article, or claim that may be made by its manufacturer, is not guaranteed or endorsed by the publisher.
